# Mind the Gaps in Tumor Immunity: Impact of Connexin-Mediated Intercellular Connections

**DOI:** 10.3389/fimmu.2017.01067

**Published:** 2017-09-01

**Authors:** María Alejandra Gleisner, Mariela Navarrete, Francisca Hofmann, Flavio Salazar-Onfray, Andrés Tittarelli

**Affiliations:** ^1^Disciplinary Program of Immunology, Faculty of Medicine, Institute of Biomedical Sciences, Universidad de Chile, Santiago, Chile; ^2^Faculty of Medicine, Millennium Institute on Immunology and Immunotherapy, Universidad de Chile, Santiago, Chile

**Keywords:** gap junction, connexin, tumor immunity, tumor microenvironment, stromal cells

## Abstract

Gap junctions (GJs)-mediated intercellular communications (GJICs) are connexin (Cx)-formed plasma membrane channels that allow for the passage of small molecules between adjacent cells, and are involved in several physiopathological processes, including immune responses against cancer. In general, tumor cells are poorly coupled through GJs, mainly due to low Cx expression or reduced channel activity, suggesting that Cxs may have tumor suppressor roles. However, more recent data indicate that Cxs and/or GJICs may also in some cases promote tumor progression. This dual role of Cx channels in tumor outcome may be due, at least partially, to the fact that GJs not only interconnect cells from the same type, such as cancer cells, but also promote the intercellular communication of tumor cells with different types of cells from their microenvironment, and such diverse intercellular interactions have distinctive impact on tumor development. For example, whereas GJ-mediated interactions among tumor cells and microglia have been implicated in promotion of tumor growth, tumor cells delivery to dendritic cells of antigenic peptides through GJs have been associated with enhanced immune-mediated tumor elimination. In this review, we provide an updated overview on the role of GJICs in tumor immunity, focusing on the pro-tumor and antitumor effect of GJs occurring among tumor and immune cells. Accumulated data suggest that GJICs may act as tumor suppressors or enhancers depending on whether tumor cells interact predominantly with antitumor immune cells or with stromal cells. The complex modulation of immune-tumor cell GJICs should be taken into consideration in order to potentiate current cancer immunotherapies.

## Introduction

Gap junctions (GJs) are intercellular channels found at the plasma membrane that allow direct communication between adjacent cells. Functional GJs are composed of connexin (Cx) proteins. Cxs form hexameric hemichannels (Cx-HCs) inserted into the membrane of one cell, which then docks with a Cx-HC from an adjacent cell to establish a GJ channel (1). When Cx-HCs acquire an open conformation, they allow for the bidirectional exchange of molecules between the cytoplasm and the extracellular fluid. The Cx gene family is comprised of 21 members in humans and 20 members in mice, and they are usually named after their predicted molecular weight, for example Cx43 corresponds to a Cx of 43 kDa. Whereas most Cx isoforms are strictly expressed in a tissue-specific manner, Cx43 is expressed almost ubiquitously, and is the main Cx member in the immune system (2). Each Cx-HC can be formed by one or more isoform of Cx proteins, which determines, at least partially, GJ permeability and their regulatory properties (1). GJs and Cx-HCs allow for the intercellular passage and the intake/uptake from or to the extracellular fluid of small (~1.4 nm) and immunologically relevant molecules, including adenosine triphosphate (ATP), cyclic adenosine monophosphate (cAMP), uridine diphosphate (UDP), cyclic guanosine monophosphate–adenosine monophosphate (cGAMP), inositol triphosphate (IP_3_), Ca^2+^, microRNAs (miRNAs), and small peptides (3).

Gap junction-mediated intercellular communications (GJICs) are critical for several physiological processes, including: electric current propagation in the heart and neurons (4, 5); embryonic development (6); cell differentiation (7); tissue homeostasis (8); autophagosome biogenesis (9); cell survival, proliferation, and cell death (10, 11); and the immune response (12). As GJs are involved in countless cellular and physiological processes, the cells need to establish delicate regulatory mechanisms of GJICs, which occurs at different levels, such as that of Cx gene expression, the life cycle of Cx protein level, or GJ assembly and permeability. These different mechanisms of GJIC regulation are at the same time highly responsive to environmental cues, including pro-inflammatory signals. Excellent reviews about regulatory mechanisms of Cx expression and GJIC have been recently published (13–21).

Mutations in Cx genes or loss of Cx channel functionality have been implicated in the development of different diseases, such as congenital deafness (22), skin disorders (23), cardiac arrhythmias (24), cataracts (25), and cancer (26). The role of Cx channels in the incidence and progression of cancer has been extensively investigated since the year 1966 when Loewensteind and Kanno showed that the electrical coupling found in normal hepatocytes was lost in liver tumors (27). A substantial progress in our understanding of GJ-mediated cell coupling in cancer has occurred since then, and it was recently reviewed (26, 28, 29). In general, cancer cells derived from various tumor types show reduced expression of Cxs and low GJ cell coupling, leading to the concept that Cxs are tumor suppressor genes, principally due to the antiproliferative effect of their overexpression in tumor cells (30). However, recent evidence also indicates that this is partially true and depends on cancer type, disease stages, and Cx isotype (28). Indeed, an analysis of different clinical studies for 15 different cancer types indicates that the expression of Cxs in tumor biopsies could be associated with good or bad prognosis of cancer progression, depending on the Cx isotype and the type of cancer (Table 1).

**Table 1:** Associations between connexin (Cx) expression in tumors and clinical outcome in cancer patients.

Cancer type	Cx	Clinical manifestation	Reference
Prostate	Cx43	High BRFS	Xu et al. (31)
	Cx26^a^	Low metastasis	Bijnsdorp et al. (32)
	Cx43	High OS	Benko et al. (33)
ESCC	Cx43	High OS	Tanaka et al. (34)
	Cx26	High LNM; low FYS	Inose et al. (35)
GCTB	Cx43	High PFS	Balla et al. (36)
NMIUBC	Cx43	Low PFS	Poyet et al. (37)
Breast	Cx43	High RDFS	
	Cx30	Low RDFS	
	Cx26	High RDFS	
	Cx32	Low RDFS	Teleki et al. (38)
	Cx26	Low OS after chemotherapy	
	Cx46	High OS after chemotherapy	Teleki et al. (39)
	Cx26	High LNM	Naoi et al. (40)
	Cx26	High recurrence 5 year	
	Cx43	Low OS	Stoletov et al. (41)
OSCC	Cx43	Low OS	Brockmeyer et al. (42)
Gastric	Cx43	Low LNM	Tang et al. (43)
	Cx26	High OS	Liu et al. (44)
NSCL	Cx43	High OS after chemotherapy	Du et al. (45)
	Cx43^a^	Low nodal micrometastasis	Chen et al. (46)
LSCC	Cx26	Low FYS	Ito et al. (47)
GBM	Cx46^b^	High OS	Hitomi et al. (48)
HNSCC	Cx43	High OS	Dános et al. (49)
HCC	Cx43	High OS	Wang et al. (50)
Colorectal	Cx43	High OS	Sirnes et al. (51)
	Cx26	High OS	Nomura et al. (52)
	Cx26	Low DFS and LMFS	Ezumi et al. (53)
Melanoma	Cx26	High metastasis	Haass et al. (54)
	Cx26	Low OS	
	Cx43	Low OS, high metastasis	Stoletov et al. (41)
Pancreatic	Cx43	Low LNM	Liang et al. (55)
	Cx26	Low OS	Zhu et al. (56)

*BRFS, biochemical recurrence-free survival; DFS, disease-free survival; ESCC, esophageal squamous cell carcinoma; FYS, five-year survival; GBM, glioblastom multiform; GCTB, giant cell tumor of bone; HCC, hepatocellular carcinoma; HNSCC, head and neck squamous cell carcinoma; LMFS, lung metastasis-free survival; LNM, lymph node metastasis; LSCC, lung squamous cell carcinoma; NMIUBC, non-muscle invasive urothelial bladder cancer; NSCL, non-small-cell lung; OS, overall survival; OSCC, oral squamous cell carcinoma; PFS, progression-free survival; RDFS, relapse/disease-free survival*.

*In green: Cx expression is associated with an antitumoral effect; in red: Cx expression is associated with a pro-tumoral effect*.

*^a^Cx expression evaluated in normal adjacent cells*.

*^b^Cx expression evaluated in cancer stem cells*.

Moreover, accumulated evidences strongly suggest that diverse aspects of the functionality of Cxs could differentially impact tumor progression: (i) besides their role as channel forming units, Cxs have channel-independent activities that may affect tumor cell growth (29, 57); (ii) Cx-HCs have differential roles than Cx-GJs in cancer cells (58); and (iii) Cxs can mediate the formation of homotypic GJICs among tumor cells, and/or the formation of heterotypic GJICs between different cell types within the tumor microenvironment, which could differentially impact the tumor cells fate. In this review article, we summarize recent data describing the pro- and antitumor effects of the heterotypic GJICs that tumor cells form (either with immune and non-immune cells), focusing on the role of GJICs in the antitumor immune response. A large number of evidences suggest that Cx expression on tumor cells may promote or halt cancer progression depending on the type of cells engaged at the tumor microenvironment. In general, tumor GJICs with immune cells may promote responses against tumors, while tumor interactions with some stromal cells through GJs may inhibit or enhance tumor cell growth depending on the particular context where those interactions occur.

## Heterotypic GJICs among Cancer Cells and Immune Cells: Role of GJ in Antitumoral Immunity

**Figure 1:**
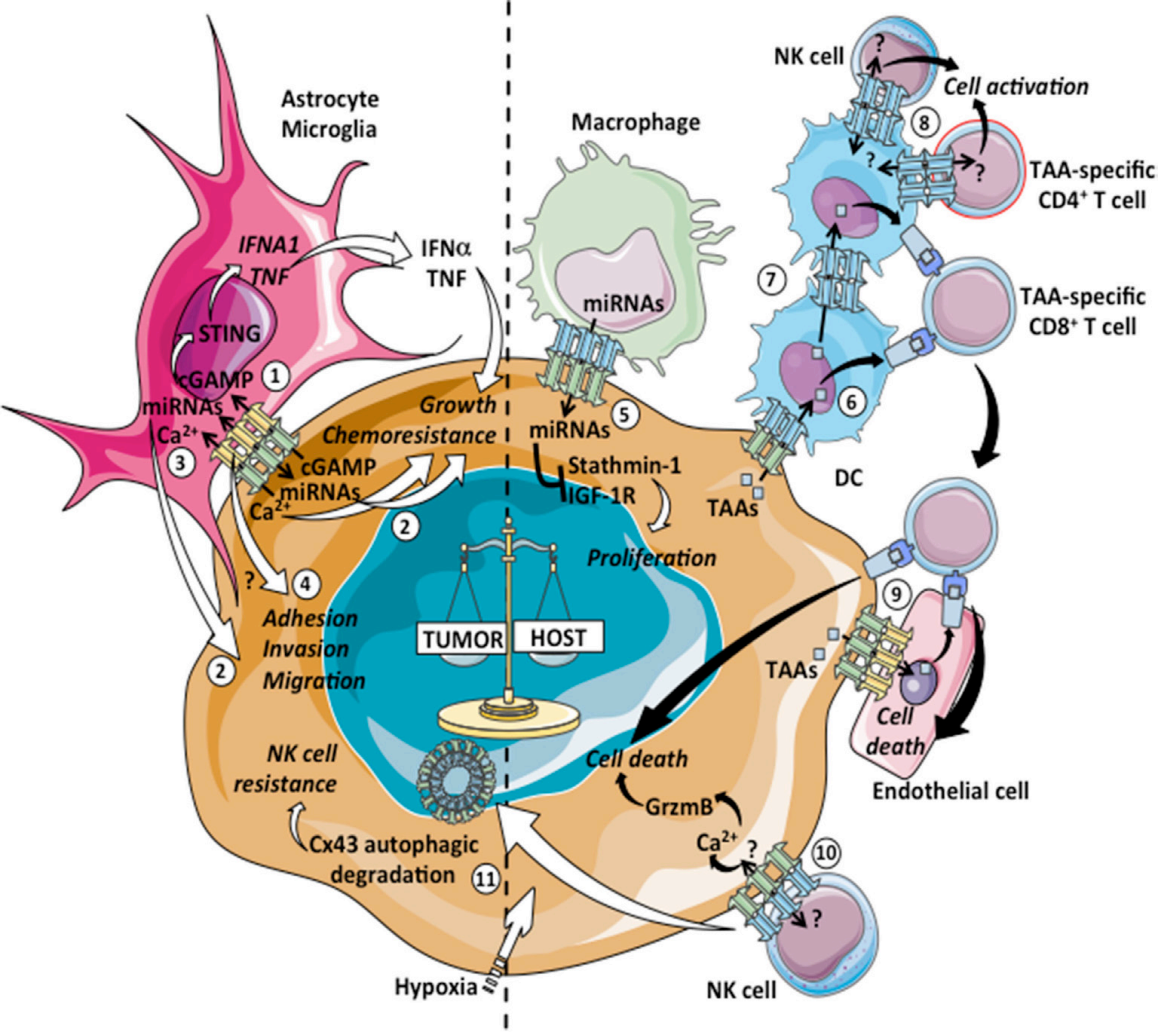
Pro- and antitumoral effects of tumor-immune cell heterotypic GJICs. Gap junction (GJ)-mediated communications among tumor cells and immune cells can lead to pro-tumoral (left: 1–4, 11) or antitumoral (right: 5–10) consequences. 1: carcinoma–astrocyte interactions promote brain metastasis of breast and lung cancers through the passage of the second messenger cyclic guanosine monophosphate–adenosine monophosphate (cGAMP) from tumor cells to astrocytes *via* Cx43-GJs, the subsequent activation of the STING pathway and the production of IFN-α and TNF that act as paracrine signals promoting growth and chemoresistance of tumor cells. 2: GJ-mediated diffusion of pro-survival microRNAs (miRNAs) between mouse astrocytes and human lung cancer cells provides increased resistance to chemotherapy. Similarly, the transfer of miRNAs from glioma to astrocytes induces glioma invasion. 3: astrocytes reduce apoptosis in melanoma cells treated with different chemotherapeutic drugs by sequestering intracellular Ca^2+^ *via* GJICs. 4: GJICs among glioblastoma cells and astrocytes contribute through uncharacterized mechanisms to the adhesion, migration, and invasion of tumor cells to the brain parenchyma. 5: GJ-mediated transfer of miRNAs from macrophages to hepatocellular carcinoma cell lines regulates gene expression of stathmin-1 and insulin-like growth factor-1 receptor and inhibits tumor cell proliferation. 6: Cx43 expression in melanoma cells allows for the transfer of preprocessed tumor associated antigens (TAAs) from melanoma cells to dendritic cells (DCs), improving DC-based tumor vaccination by increasing CD8^+^ T cell activation and antitumor immunity. 7: Cx43-GJs participate in melanoma antigen transfer and cross-presentation between human DCs, facilitating a more effective DC-mediated CD4^+^ T cell activation. 8: Cx43-GJs accumulate at the immunological synapse (IS) formed between DCs and melanoma-specific CD4^+^ T cells and natural killer (NK) cells, contributing to cell activation. 9: Cx43-GJs allow for the passage of TAA peptides from melanoma to autologous endothelial cells, inducing their cross-recognition and elimination by TAA-specific CD8^+^ T cells. 10: Cx43-GJs accumulate at the lytic IS formed between NK cells and melanoma cells, contributing to Ca2^+^ influx and granzyme-b (GrzmB)-mediated induction of apoptosis in the target cells. 11: Activation of autophagy in hypoxic melanoma cells causes the selective degradation of GJ-Cx43, impairing NK cell-mediated tumor cell killing.

Almost all immune cells and their hematopoietic precursors express Cx proteins, and nowadays its ability to modulate different aspects of immune responses is well recognized (2, 12, 59, 60). Cx channels and GJICs have been implicated in hematopoiesis, hemostasis, phagocytosis, immune cell migration, lymphocyte responses, antigen (Ag) cross-presentation, inflammation, immune tolerance, and cancer immunity. In Table 2, we summarize the current information available on the role of Cxs, Cx-HCs, and GJICs in the immune system activities. Here, we summarize recent data describing immune cell–cancer cell heterotypic GJICs that negatively impact tumor progression (Figure 1, right panel).

**Table 2:** Summary of the role of connexin (Cx) channels in immunity.

Immunological process	Role of Cx channels	Reference
Hematopoiesis	Cx43 expression is required for late stages of primary T and B lymphopoiesis during embryogenesis	Montecino-Rodriguez et al. (61)
	Cx43 and Cx32 expression is necessary for hematopoietic regeneration after 5-FU cytoablative treatments	Montecino-Rodriguez et al. (61); Presley et al. (62); Taniguchi Ishikawa et al. (63); Hirabayashi et al. (64)
	Stromal functional Cx43-GJs contribute to stromal regulation of the clonal outgrowth of HP in fetal liver	Cancelas et al. (65)
	Cx43 regulates HSC/P proliferation and differentiation of myeloid blood cell precursor cells	Bodi et al. (66); Flenniken et al. (67)
	Cx32 regulates cell proliferation and content of HP in the BM	Hirabayashi et al. (64)
	GJs allow the IL-3/GM-CSF-dependent intracellular Ca^2+^ raise required for hematopoiesis	Paredes-Gamero et al. (68)
	Cx43 controls the cellular content of BM osteogenic microenvironment and is required for homing of HSCs in myeloablated animals	Gonzalez-Nieto et al. (69)
	Cx43 reduces senescence of HSCs by regulating ROS content *via* ROS transfer to the BM hematopoietic microenvironment during stress-induced hematopoietic regeneration	Taniguchi Ishikawa et al. (63)
	Cx43- and Cx45-GJs regulate CXCL12 secretion by BMSC and homing of HSC and leukocytes to the BM	Schajnovitz et al. (70)
Hemostasis and thrombosis	Cx37-GJIC between aggregating platelets limits thrombus propensity by downregulating platelet reactivity	Angelillo-Scherrer et al. (71)
	Cx37 and Cx40 channels participate in platelet aggregation, fibrinogen binding, granule secretion, and clot retraction	Vaiyapuri et al. (72); Vaiyapuri et al. (73)
Immune tolerance/Treg cell activity	GJ-mediated transfer of cyclic adenosine monophosphate (cAMP) is involved in Treg cell-mediated suppression of responder T cells	Bopp et al. (74)
	GJIC between Treg cells and DCs abrogates the *de novo* induction of CD8^+^ T responses during the sensitization phase of experimental CHS reactions by interfering with T cell stimulatory activity of DCs	Ring et al. (75)
	Expression of Cx43 in thymic Treg cell progenitors supports Treg cell development	Kuczma et al. (76)
	GJ-mediated cAMP transfer from Treg cell to DCs controls GvHD	Weber et al. (77)
	Cx43-GJIC is a component of the Treg cell suppression mechanism compromised in aging NOD mice	Kuczma et al. (78)
Inflammation/Immune cells migration	GJ coupling between neutrophils and the endothelium favors transmigration of neutrophils and modulates leakiness	Zahler et al. (79)
	Acinar Cx32-GJIC modulates the severity of acute pancreatitis	Frossard et al. (80)
	GJs favor monocyte/MØ transmigration across a BBB *in vitro* model. TNF-α/IFN-γ-stimulated monocyte/MØs secrete MMP-2 in a GJ-dependent manner	Eugenín et al. (81)
	Cx43 channels participate in atherosclerotic plaque formation *in vivo*	Kwak et al. (82); Wong et al. (83)
	Cx43 expression in wounded skin promotes inflammation and retard wound closure time *in vivo*	Qiu et al. (84)
	ATP released *via* Cx43 channels of activated neutrophils modulates endothelial cell function during inflammation	Eltzschig et al. (85)
	ATP released *via* Cx37 channels of monocytes inhibits their adhesion to the endothelium, controlling the initiation of atherosclerotic plaques	Wong et al. (86)
	Endothelial Cx43 and GJIC allow leukocyte adhesion and transmigration during acute inflammation *in vivo*	Véliz et al. (87)
	Cx43-GJIC between fibroblasts and mast cells promotes fibroblast pro-fibrotic activities	Pistorio and Ehrlich (88)
	Cx43-GJs participates in eosinophils transendothelial migration	Vliagoftis et al. (89)
	Cx43-GJs are positive regulators of B cell motility, CXCL12-directed migration and transendothelial migration	Machtaler et al. (90)
Infection immunity	Cx43 participates in MØ phagocytosis activity and plays a protective role in host survival in response to *E. coli*-induced peritonitis	Anand et al. (91)
	GJICs are necessary for the amplification of IRF3 pathway activation and the propagation of antiviral and inflammatory responses in response to cytosolic dsDNA	Patel et al. (92)
	Cx43-GJs allow cell-to-cell propagation of NFκB and MAP kinase pro-inflammatory pathways from *S. flexneri-, L. monocytogenes-*, or *S. typhimurium*-infected to uninfected epithelial cells, leading to IL-8 production by bystander cells	Kasper et al. (93)
	*S. epidermidis*-derived PGN induces Cx43-HCs and GJ coupling in endothelial cells. ATP released by Cx43-HCs induces IL-6 and TLR2 expression in PGN-stimulated epithelial cells	Robertson et al. (94)
	Treg cells control HIV replication in conventional autologous T cells *via* a Cx43-GJ/cAMP-dependent mechanism	Moreno-Fernandez et al. (95)
	GJICs mediate the transfer of cGAS-triggered cGAMP from DNA virus- or *C. trachomatis*-infected to bystander non-infected cells, leading to the propagation of type I IFN signaling	Ablasser et al. (96); Zhang et al. (97)
	LPS-induced Cx43 channels protect mice against *E. coli* infection *via* the release of the extracellular danger signal UDP	Qin et al. (98)
CNS immunity	Astrocytic Cx43-GJs play a neuroprotective role during ischemia, regulating the apoptosis and the inflammatory response after stroke	Nakase et al. (99)
	Release of glutamate *via* Cx-HCs of activated microglia triggers neuronal death during inflammation, ischemia or autoimmune encephalomyelitis	Takeuchi et al. (100); Takeuchi et al. (101); Shijie et al. (102)
	Cx43 channels participate in the metabolic status of astrocytes during inflammation	Retamal et al. (103)
	Astrocytes reduce apoptosis of melanoma cells treated with different chemotherapeutic drugs by sequestering intracellular Ca^2+^ *via* GJs	Lin et al. (104)
	Inflammation or hypoxia-induced astroglial Cx43-HC activation induces neuronal and astroglial cell death	Froger et al. (105); Orellana et al. (106); Orellana et al. (107)
	CNS oligodendrocytes Cx47- or Cx32-GJs loss alters the CNS immune status without external triggers	Wasseff and Scherer (108)
	Astroglial Cx43 promotes immune quiescence of the brain, through setting the activated state of cerebral endothelium, which controls the immune cells recruitment and Ag presentation mechanisms	Boulay et al. (109)
	Carcinoma-astrocyte Cx43-GJs promote brain metastasis by cGAMP transfer	Chen et al. (110)
	Lung cancer cells acquired pro-survival miRNAs from astrocytes in a GJ-dependent manner	Menachem et al. (111)
Mucosal immunity	GJs coordinate ciliary beating in respiratory mucosa airway cells	Sanderson et al. (112); Boitano et al. (113); Homolya et al. (114)
	Cx43-GJs spread Ca^2+^-dependent pro-inflammatory signals in the lung capillray bed	Parthasarathi et al. (115)
	*S. flexneri*-induced Cx26-HC opening promotes signaling events leading to bacterial invasion and dissemination in gastrointestinal epithelial cells	Tran Van Nhieu et al. (116); Romero et al. (117); Simpson et al. (118)
	TLR2-induced GJICs amplify pro-inflammatory signaling by communicating Ca^2+^ fluxes from *P. aeruginosa*-infected to adjacent bystander airway epithelial cells thus increasing CXCL8 secretion and neutrophils recruitment to the infected lungs	Martin and Prince (119)
	Cx43-GJs favor neutrophils transmigration to the lungs after intra-traqueal instillations of *P. aeruginosa* LPS	Sarieddine et al. (120)
	*C. rodentium* infection induces Cx43 expression and Cx43-HC opening in the apical membranes of infected colonocytes, contributing to the generation of diarrhea during infectious enteric disease	Guttman et al. (121)
	TLR2-induced Cx43-GJICs maintain intestinal epithelial barrier during acute and chronic inflammatory injury	Ey et al. (122)
	Cx40-GJIC contributes to a quiescent non-activated endothelium by propagating adenosine-evoked anti-inflammatory signals between endothelial cells	Chadjichristos et al. (123)
	GJICs coordinate the signaling cascade leading to airway surface liquid secretion	Scheckenbach et al. (124)
	Intestinal epithelial cells release ATP *via* Cx-HCs as an early alert response to *S. flexneri* infection, which promotes inflammation of the gut	Puhar et al. (125)
	Cx43-GJIC is necessary for innate immune activation by regulating the survival/apoptosis balance of airway epithelial cells in response to *P. aeruginosa*	Losa et al. (126)
	Establishment of oral tolerance *via* Cx43-GJ-mediated transfer of fed Ags from gut MØs to DCs	Mazzini et al. (127)
	Alveolar MØs establish Cx43-GJIC with the epithelium through synchronized Ca^2+^ immunosuppressive wave signals to reduce endotoxin-induced lung inflammation	Westphalen et al. (128)
	TLR ligands induce GJIC between sentinel globet cell guards in the colonic crypt favoring mucin2 secretion	Birchenough et al. (129)
APC and lymphocyte activity	Cx43-GJs communicates FDCs with FDCs and with B cells in germinal centers and support FDC-B cell cluster formation and cell survival	Krenacs et al. (130); Rajnai et al. (131)
	Cx40- and 43- but not Cx26-, 32-, 37- nor 45-GJICs are present in peripheral blood and tonsil T, B, and NK lymphocytes; their expression are induced by PHA and LPS and participates in the secretion of IL-10, IgM, IgG and IgA in mixed lymphocytes cocultures	Oviedo-Orta et al. (132); Oviedo-Orta et al. (133)
	Cx43-GJIC allows cross-presentation of influenza-derived Ag peptides between influenza-infected Cx43-transfected human squamous or primary HUVEC endothelial cells and human primary IFN-γ/TNF-α-stimulated monocytes	Neijssen et al. (134)
	Cx43-GJIC between murine BMDCs or DC cell line is required for effective LPS/IFN-γ-mediated activation of DCs	Matsue et al. (135)
	Melanoma cell lysate-pulsed/TNF-α stimulated MDCs transfer and cross present melanoma derived Ag peptides between MDCs by Cx43-GJs	Mendoza-Naranjo et al. (136)
	Cx43-GJ allows the cross-presentation of Ag peptides from live or apoptotic tumor cells to DCs or endothelial cells	Pang et al. (137); Benlalam et al. (138); Saccheri et al. (139)
	Cx43-GJs and HCs are localized in the murine and human DC-T Cell immunological synapse (IS) in an Ag-dependent fashion and are required for DC-mediated T cell activation	Elgueta et al. (140); Mendoza-Naranjo et al. (141); Yu et al. (142)
	Cx43-HCs are required by CD4^+^ T cells for sustain their clonal expansion after Ag recognition	Oviedo-Orta et al. (143)
	Cx43 regulates B lymphocyte spreading and adhesion	Machtaler et al. (144)
	Anti-proliferative miRNAs are transferred from human MØs to hepatocarcinoma cells *via* GJs	Aucher et al. (145)
	Cx43-GJs are localized in the human DC-NK and NK-tumor cell ISs and Cx43-GJIC is required for DC-mediated NK cell activation and NK cell-mediated tumor cell lysis	Tittarelli et al. (146); Tittarelli et al. (147)

*5-FU, 5-fluorouracil; Ags, antigens; APC, professional Ag presenting cell; BBB, blood–brain barrier; ATP, adenosine triphosphate; BM, bone marrow; BMDC, bone marrow-derived DC; BMSC, bone marrow stromal cells; cGAMP, cyclic guanosine monophosphate (GMP)–adenosine monophosphate (AMP); cGAS, cyclic GMP-AMP synthase; CHS, contact hypersensitivity; CNS, central nervous system; *C. rodentium, Citrobacter rodentium*; *C. trachomatis, Chlamydia trachomatis*; DC, dendritic cell; *E. coli, Escherichia coli*; FDC, folicular dendritic cells; GJ, gap junction; GJIC, gap junction-mediated intercellular communication; GvHD, graft-versus-host disease; HC, hemichannel; HIV, human immunodeficiency virus; HP, hematopoietic progenitors; HSC/P, hematopoietic stem cells/progenitors; HUVEC, human umbilical vein endothelial cell; IFN, interferon; IL, interleukin; IRF, IFN responsive factor; *L. monocytogenes, Listeria monocytogenes*; LPS, lipopolysaccharide; MØ, macrophages; MDC, monocyte-derived DC; miRNA, microRNA; MMP-2, metalloproteinase-2; NK, natural killer cell; NOD, non-obese diabetic; *P. aeruginosa, Pseudomonas aeruginosa*; PGN, peptidoglycan; PHA, phytohemagglutinin; ROS, reactive oxygen species; *S. epidermidis, Staphylococcus epidermis*; *S. flexneri, Shigella flexneri*; *S. typhimurium, Salmonella typhimurium*; TLR, toll-like receptor; TNF, tumor necrosis factor; UDP, uridine diphosphate*.

Initial studies exploring a potential antitumoral role of GJICs among cancer cells and immune cells were encouraged by the seminal work of Jacques Neefjes and collaborators in 2005, where the transfer and cross-presentation of viral Ag peptides *via* GJs was reported (134). In this study, it was shown that GJ-negative human squamous carcinoma cells (A431 cell line) transferred micro-injected 9-mer linear peptides to surrounding non-micro-injected cells only when the cells were stably transfected with the human Cx43 gene. The closure of GJs by 2-aminoethoxydiphenyl borate prevented this intercellular peptide transfer. Neijssen and coworkers also evaluated the Cx43-GJ-mediated transfer of endogenous and immunologically relevant Ag peptides. They showed that human primary human leukocyte Ag (HLA)-A2^+^ monocytes stimulated with interferon (IFN)-γ and tumor necrosis factor (TNF)-α (cytokines that induce Cx43 expression) efficiently acquired influenza-derived Ag peptides (FluM_57–65_) from influenza-infected cells (A431 or endothelial cells) *via* Cx43-GJs, allowing for the subsequent monocyte-mediated cross-priming of an HLA-A2-restricted FluM_57–65_-specific T-cell clone (134).

Later, our group described that melanoma Ag peptides could also be transferred and cross-presented between human dendritic cells (DCs) *via* Cx43-GJs (136). In this work, we reported that melanoma patient’s HLA-A2^-^ monocyte-derived DCs incubated overnight with an allogeneic melanoma cell lysate (MCL), efficiently transfer MelanA/MART1_27–35_ peptides to HLA-A2^+^ monocyte-derived DCs, leading to the subsequent activation of an HLA-A2-restricted MelanA/MART1_27–35_-specific cytotoxic T lymphocyte (CTL) clone (136). The transfer of MelanA/MART1_27–35_ peptides between DCs was strongly prevented by two different GJ chemical inhibitors (oleamide and 18β-glycyrrhetinic acid) or by a Cx43 inhibitor mimetic peptide, indicating the involvement of Cx43-GJs in the cross-presentation of tumor associated Ags (TAAs) in human DCs. Of note, in a series of clinical trials, these MCL-DCs were used as an antitumor immunotherapy for advanced malignant melanoma patients (148–153). In this series of studies, a positive correlation between the immune response induced by MCL-DC-vaccination, as established by a patient tumor-specific delayed-type IV hypersensitivity reaction and improved long-term survival was reported. In this context, it has been suggested that the efficient clinical effect of adoptively transferred DC vaccines may be improved by their potential to interact with local DCs *in vivo* and/or other cell types in peripheral tissues and lymph nodes (154). These interactions could include GJ-mediated Ag transfer and cross-presentation from injected DCs to local DCs, which may finally amplify the Ag-specific DC-mediated T cell activation. Indeed, it was reported in a murine model, that the OVA_257–264_ Ag peptide transfer from OVA-expressing DC vaccines to endogenous professional Ag presenting cells (APCs) was required for efficient OVA_257–264_-specific CD8^+^ T cell priming (155). Although in these studies the GJ-mediated transfer of Ag peptides was not evaluated, the group of Rescigno, in a murine model of oral tolerance to fed Ags, elegantly showed that Cx43-GJ-mediated transfer of Ag peptides between gut resident APCs occurs *in vivo* (127). These observations strongly suggest that the spreading of Ag peptides between cells by GJs could be a general mechanism of Ag cross-presentation.

In the context of tumor immunity, very interesting findings suggest that Cx43-mediated transfer of Ag peptides from melanoma cells to DCs could be the major mechanism of tumor Ag cross-presentation occurring *in vivo* (139). This mechanism allows DCs infiltrating *Salmonella*-infected Cx43 positive melanoma tumors to acquire preprocessed Ag peptides from the cancer cells, leading to the activation of tumor Ag-specific CTLs that finally eliminate distal tumors. The authors showed that this antitumor immune response against distal tumors was strongly abrogated when the *Salmonella*-treated tumor cells were silenced for Cx43 (139). Moreover, DC vaccines loaded *ex vivo* with *Salmonella*-infected B16 melanoma cells were more efficient in inducing melanoma growth inhibition in vaccinated mice compared to other types of DC vaccines, but only when Cx43 was not silenced in the *Salmonella*-infected B16 cells used for loading DC vaccines. These results indicate that transfer of TAA peptides from tumor cells to DCs through Cx43-GJs is far more effective than standard pathways of DC Ag-loading in generating protective DC-based vaccines (139). Interestingly, DCs can also acquire tumor-derived Ag peptides by GJ coupling with apoptotic tumor cells, as suggested by the findings of Pang and coworkers (137). This could be of great relevance in the immune response against tumors, since caspase activation can expose neo epitopes in early apoptotic tumor cells through the direct cleavage of proteins, which results in epitopes from these proteins being favored for cross-presentation, and thus amplifying the repertoire of cross-presented Ags (156). Additionally, in a 3D *in vitro* cell coculture model, Cx43-GJs have been implicated in the Ag peptide transfer from melanoma to autologous endothelial cells. Once endothelial cells acquire the melanoma Ag peptides, they become susceptible to cross-recognition and elimination by an autologous tumor-specific CTL clone (138). In fact, Cx43-GJs can be detected among melanoma and endothelial cells in metastatic biopsies from patients (157), suggesting that CTL-mediated elimination of endothelial cells may contribute to control tumor progression, which needs further investigation.

In addition to peptides, the GJ-mediated transfer of miRNAs between tumor cells and immune cells have been implicated in tumor immunity. Specifically, Aucher and collaborators (145) reported that miR-142 and miR-223, which are endogenously expressed in human macrophages (MØs) but not in hepatocarcinoma cells (HCCs), were transferred from MØs to HCC cells *via* GJs and effectively target the expression of stathmin-1 and insulin-like growth factor-1 receptor in the acceptor tumor cells leading to the inhibition of tumor cell proliferation.

Furthermore, recent evidences suggest that Cx43 is a component of the immune synapse, and that Cx43-GJICs are required for Ag-dependent DC-mediated T cell activation (140, 141). In this context, we reported that Cx43 channels (both GJs and HCs) accumulate at the immunological synapse (IS) during DC-mediated Ag-specific CD4^+^ T cell priming, mediating the bidirectional crosstalk between DCs and T cells. This phenomenon was observed in both murine (DC-OVA/OT-II T cells) and human (MCL-DCs/melanoma-specific autologous CD4^+^ T cell clone) models. The evidence indicated that Cx43-GJICs between DCs and T cells regulates Ca^2+^ signals and DC-mediated T cell activation (141), pointing to a role for Cx43 as an important functional component for intercellular signaling in the immune system. Similarly, Cx43 accumulation was detected at the interface of mature human DCs and autologous resting natural killer (NK) cells, mediating bidirectional GJICs between these cells. The blockade of Cx43-GJs strongly inhibits the DC-mediated activation of NK cells, as measured by NK cell expression of CD69 and CD25 and the secretion of IFN-γ (146). The nature of the molecules shuttled *via* Cx43 channels at the IS between DCs and lymphocytes (both T cells and NK cells) remains uncharacterized, but as lymphocyte activation requires ATP and Ca^2+^ for biomass synthesis and signal transduction (158, 159), both molecules are reasonable candidates to be mobilized from DCs to lymphocytes by Cx43-GJs.

Moreover, Cx43 channels seem to accumulate at the interface of NK cells and target tumor cells (myelogenous leukemia or melanoma cells) and to mediate intercellular communications that participate in NK cell-mediated tumor cell lysis (146, 147). Cx43-GJICs among NK cells and tumor cells appear to not affect tumor-induced NK cell degranulation but instead do control the NK cell cytotoxicity by contributing to granzyme-b activity and Ca^2+^ influx into tumor cells (146, 147). Moreover, Cx43 expression in target tumor cells renders these cells more susceptible to NK cell-mediated lysis. Indeed, Cx43 gene knockdown in Cx43 positive tumor cells decreases the level of NK cell-mediated lysis to the same extent as the prevention of GJICs by chemical inhibitors or Cx43 mimetic peptides (146). Additionally, different melanoma cell lines or MCF-7 breast cancer cells with low or negative expression of Cx43, showed diminished susceptibility to NK cell-mediated lysis compared to the Cx43-overexpressing counterparts (147). Additional data, indicating the importance of Cx43 at the NK cell/tumor cell lytic IS, were obtained from the evaluation of the regulation of Cx43 by tumor hypoxia. Hypoxic stress, frequently occurring in the microenvironment of solid tumors, is involved in the tumor escape of immune surveillance, including a suppressed susceptibility of tumor cell lysis by CTLs and NK cells (160). While hypoxic stress increased the total Cx43 protein level in a hypoxia-induced factor 1α-driven manner in melanoma cells, the presence of Cx43 channels at the IS between hypoxic melanoma cells and NK cells was strongly diminished (147). The decline of Cx43 channels at the lytic IS was dependent on increased autophagic flux occurring during hypoxia. Indeed, the presence of Cx43 at the IS could be restored in hypoxic melanoma/NK cell cocultures by inhibiting hypoxia-induced autophagy flux by hydroxychloroquine or the hypoxia-induced autophagosome formation by 3-methyladenine or knock down of the *ATG5* gene in hypoxic melanoma cells (147). Importantly, the inhibition of hypoxia-induced autophagy and thus the prevention of the subsequent autophagy-mediated degradation of Cx43 at the lytic IS was very effective in restoring the susceptibility of hypoxic melanoma cells toward NK cell-mediated lysis. These findings were corroborated using the endocytic Cx43^Y286A^ mutant (161), which remained present at the lytic IS during hypoxic stress and restored the susceptibility of melanoma cells to lysis by NK cells in hypoxic conditions, which is inhibited by Cx43-specific inhibitory mimetic peptides (147). These reports highlight an important role for Cx43 channels at the lytic ISs among NK cells and tumor cells, and suggest that the low susceptibility of Cx43-negative tumor cells to NK cell immune surveillance is an additional mechanism that favors the survival of GJ-deficient tumor cells observed principally in primary tumors (162).

**Figure 2:**
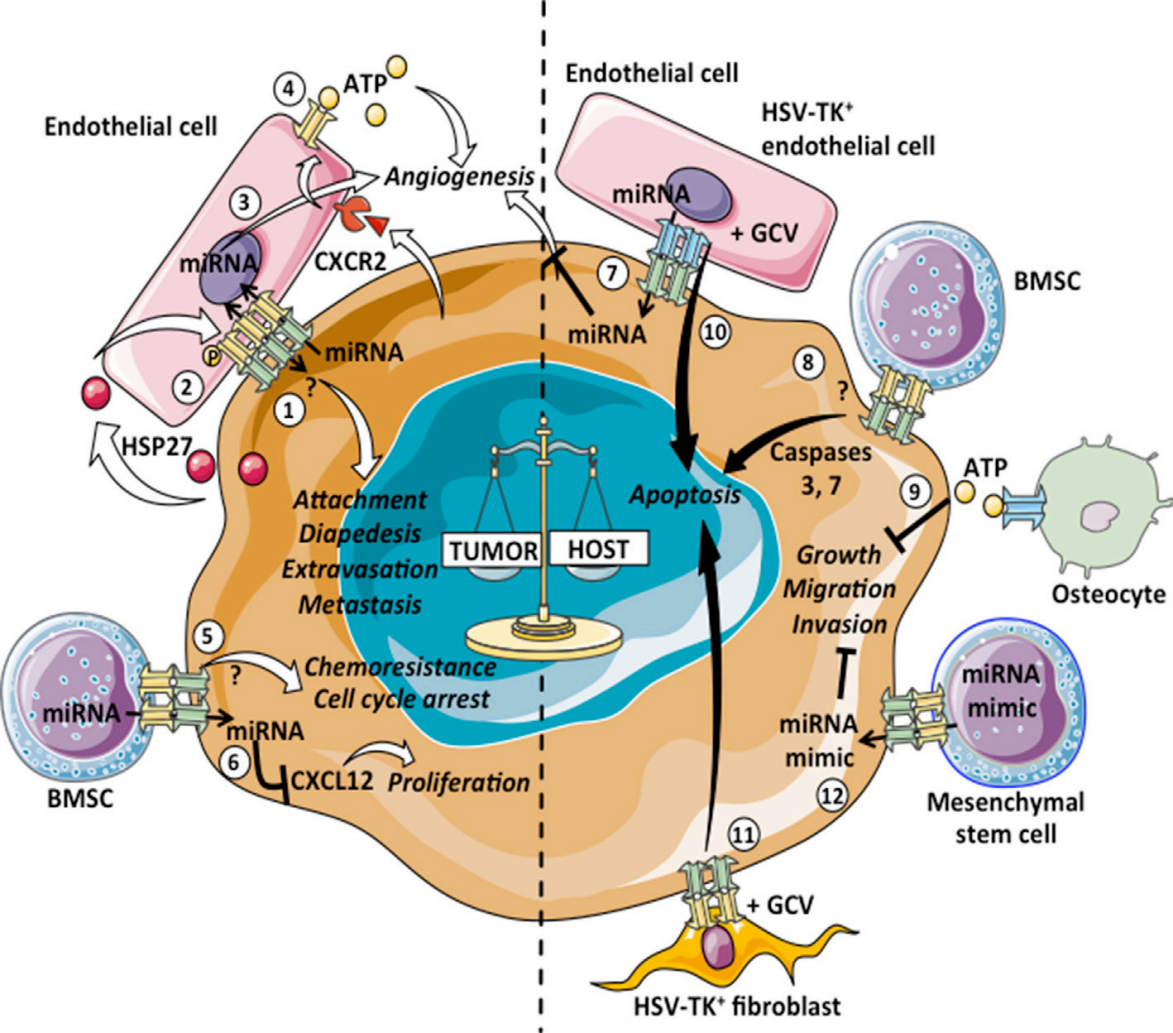
Pro- and antitumoral effects of tumor–stromal non-immune cell heterotypic GJICs. Gap junction (GJ)-mediated communications among tumor cells and normal non-immune cells can lead to pro-tumoral (left: 1–6) or antitumoral (right: 7–12) consequences. 1: intercellular communications mediated by Cx26- and Cx43-GJs among melanoma or breast cancer cells with endothelial cells promote cell attachment, diapedesis, extravasation and metastasis of tumor cells. 2: primary colon cancer cells release heat-shock protein 27 (HSP27), which induces the phosphorylation of Cx43 in endothelial cells and the subsequent formation of heterotypic GJs with tumor cells. This communication thus promotes transendothelial migration of primary colon cancer cells. 3: the GJ-mediated transfer of microRNAs (miRNAs) from glioblastoma cells to endothelial cells promotes angiogenesis. 4: metastatic colon cancer cells induce Cx32-HCs in endothelial cells *via* CXCR2. The adenosine triphosphate (ATP) released by Cx32-HCs could induce neo-angiogenesis in the metastatic foci. 5: leukemic cells GJ-coupled with bone marrow stromal cells (BMSCs) are arrested in G0 and acquire resistance to chemotherapy-induced apoptosis. 6: breast cancer cells acquired CXCL12-specific miRNAs from BMSCs *via* GJs, which induce cancer cell dormancy. 7: Cx43-GJ-mediated transfer of antitumoral miRNAs from human microvascular endothelial cells to colon cancer cells inhibits the angiogenesis induced by the cancer cells. 8: BMSCs overexpressing Cx43 form functional GJ with T lymphoblastic leukemia cells and increase the basal level of apoptosis due to the Cx43-dependent activation of caspases 3 and 7. 9: the ATP released through Cx43-HCs by osteocytes inhibits anchorage-independent growth, migration and invasion properties of human and mouse breast cancer cells. 10, 11: when herpes virus thymidine kinase (HSV-TK)^+^ endothelial cells or fibroblasts are cocultured with different tumor cell lines in the presence of ganciclovir (GCV), the extent of bystander killing correlates with the level of GJ communication between the tumor and HSV-TK^+^ cells. 12: mesenchymal stem cells deliver synthetic miRNA mimics to glioma and glioma stem cells *via* GJs, decreasing migration and self-renewal of tumor cells.

Altogether the heterotypic GJICs described so far, support a tumor suppressor role of Cxs, as its expression in tumor cells promotes a plethora of intercellular interactions between tumor cells and immune cells that limit tumor cell survival and growth *via* the induction of antitumor immune responses. In addition, malignant cells establish bidirectional communications with different stromal cells besides immune cells, such as cancer-associated fibroblast, endothelial cells, mesenchymal stem cells, bone marrow stromal cells (BMSC), and osteocytes. Heterotypic GJICs among cancer cells and their non-immune stromal cell counterparts have also been negatively associated with tumor progression (Figure 2, right panel).

## Heterotypic GJICs among Cancer Cells and Stromal Cells Associated with Antitumoral Effects

At large, GJICs between tumor cells and tumor stromal cells have been implicated in tumor inhibition. The first evidence suggesting that heterotypic GJICs between non-malignant cells and cancer cells negatively impact tumor cell growth was reported by the Loewenstein and coworkers (163). In this pioneer study, the authors showed that the growth of chemically or virally transformed malignant cells could be inhibited when those cells were chemically coupled with normal non-tumoral cells, specifically with embryo fibroblasts and rat liver cells. More recent data showed that leukemic Jurkat cells cocultured with Cx43-overexpressing BMSCs have a lower proliferation rate and higher methotrexate-induced apoptosis than Jurkat cells alone or cocultured with unmodified Cx43- and GJ-poor BMSCs (164). Similarly, BMSCs overexpressing Cx43, specifically human umbilical cord stem cells (Cx43-hUCSC), can form functional GJICs with the mouse T lymphoblastic leukemia cell line L615. The coculture of these cells increases the basal level of apoptosis in leukemic cells due to the activation of caspases 3 and 7 (165). Additionally, in a minimal residual disease mouse model, the relapse of leukemia was delayed when mice were transplanted with Cx43-hUCSC cells, suggesting a role for Cx43-mediated GJs among BMSCs and leukemic cells in the induction of tumor cell apoptosis *in vivo* (165). Another study indicates that Cx43-GJ-mediated transfer of the antitumoral miRNA miR-145-5p from human microvascular endothelial cells transfected with miR-145-5p mimics to primary colon cancer cells inhibits the cancer-induced tubulogenesis, suggesting that this heterotypic GJIC downregulate colon cancer cell growth by preventing the formation of new vessels (166). Additionally, a protective role of heterotypic communications mediated by Cx43-HCs has been described in the osteocyte-mediated suppression of breast cancer bone metastasis (167). The opening of Cx43-HCs in osteocytes induced by either bisphosphonate drugs or mechanical stimulation, allows for the release of ATP from osteocytes, which in turn inhibits anchorage-independent growth, migration, and invasion properties of human and mouse breast cancer cells. These inhibitory effects on cancer cells were attenuated when osteocytes were incubated with Cx43(E2), a specific Cx43-HC-blocking antibody. More interestingly, both Cx43 osteocyte-specific knockout mice and osteocyte-specific Δ130–136 transgenic mice with impaired Cx43-GJs and Cx43-HCs showed increased tumor growth and an attenuated inhibitory effect of bisphosphonate drugs, whereas R76W transgenic mice with functional Cx43-HCs but not Cx43-GJs in osteocytes did not show significant differences compared to control mice (167). These results indicate that heterotypic cell communications among normal and tumor cells, both *via* GJs and Cx-HCs, can mediate antitumor responses (Figure 2, right panel).

Additionally, GJICs have been associated with antitumor effects through the “bystander effect” during suicide gene therapy approaches, whereby the spread of death signals between cells occurs. Using the herpes virus thymidine kinase (HSV-TK) gene to render cancer cells sensitive to the drug ganciclovir (GCV), it was noted that HSV-TK-free neighboring tumor cells also died, and this phenomenon correlates with the level of GJs among tumor cells (168). Heterotypic GJICs have also been implicated in this kind of bystander effect. For example, when HSV-TK^+^ fibroblasts or HSV-TK^+^ endothelial cells are cocultured with different tumor cell lines, the extent of GCV-induced bystander killing correlates with the level of GJICs between tumor and HSV-TK^+^ fibroblasts or endothelial cells (169, 170). Also, heterotypic GJICs have a relevant role in other types of antitumor therapy, as reported by Lee and collaborators (171). They showed that mesenchymal stem cells, derived from different human tissues, efficiently deliver synthetic miRNA mimics to glioma and glioma stem cells *in vivo* when administered intracranially. In cocultures, it was determined that the transfer of miRNA mimics occurs *via* GJ- and exosome-dependent processes, affecting the expression of their target genes and decreasing the migration and self-renewal of glioma and glioma stem cells, respectively (171). Recently, functional Cx43 channels were identified in the membrane of exosomes and they can facilitate the release of exosomal content into target cells, including tumor cells, both *in vitro* and *in vivo* (172, 173). Indeed, the authors showed that when doxorubicin was incorporated into exosomes and used as a drug delivery vehicle to treat tumor-bearing mice, its antitumor effect was similar to the free drug regardless of the presence of Cx43 in exosomes; however, its cardiotoxicity was significantly lower when administrated in Cx43^+^ exosomes (173). This evidence strongly suggests that Cx43-GJ-mediated communications among extracellular vesicles and tumor cells could occur *in vivo*, and it is a very promising area to explore.

Altogether the heterotypic GJICs described so far, support a tumor suppressor role of Cxs, as its expression in tumor cells promotes a plethora of intercellular interactions between tumor cells and immune or non-immune stromal cells that limit tumor cell survival and growth. However, as we mentioned before, some specific heterotypic GJICs among cancer cells and their stroma have been positively associated with tumor progression.

## Heterotypic GJICs among Cancer Cells and Stromal Cells Associated with Pro-Tumoral Effects

Several groups have reported that GJ-mediated coupling between tumor cells and endothelial cells contributes to invasion and metastasis (Figure 2, left panel). For example, the B16 melanoma cell subline BL6 establishes efficient cell coupling with endothelial cells through Cx26-Cx43 heterotypic GJs, while the Cx26 negative B16 cell subline F10 does not (174). Interestingly, BL6 cells have a major spontaneous metastatic potential compared to the F10 cells. Transfections with the wild-type Cx26 render F10 cells competent for GJ coupling with endothelial cells, which in turn increases their spontaneous metastatic potential. Conversely, transfections with a dominant negative mutant of Cx26 render BL6 cells deficient in heterotypic GJ coupling and less metastatic (174). Similarly, in human melanoma lesions, melanoma cells in the invasive and perivascular areas as well as the endothelial cells of the small vessels surrounding the melanoma cell nests expressed Cx26, while melanoma cells residing in the basal layer showed lower levels of Cx26, suggesting that heterotypic GJ-mediated cell–cell adhesion and communication contributes to melanoma metastasis in humans (54, 174, 175). A role for Cx43-mediated interactions in melanoma cell diapedesis and in melanoma-endothelial cell attachment, both processes required for metastasis, has also been suggested. Villares and coworkers showed that the expression of the protease-activated receptor-1 contributes, at least partially, to the malignant phenotype of two human metastatic melanoma cell lines *via* the regulation of Cx43 expression, favoring Cx43-mediated melanoma-endothelial cell attachment (176). More recently, it was shown that Cx-mediated extravasation and heterotypic GJ formation with the brain endothelium could facilitate tumor cell integration into foreign tissues creating a more hospitable niche for metastatic growth (41). Cx26 and Cx43 expression in melanoma and breast cancer cells, respectively, contributes to the *in vivo* cell extravasation and brain microtumor formation in association with the vasculature. Interestingly, these tumor cells establish functional GJICs with endothelial cells *in vitro*, and this process seems to be necessary for spheroid formation and colonization in 3D matrices (41), suggesting that Cx43 and Cx26 mediate breast cancer cell and melanoma metastasis to the brain *via* tumor-endothelial cell GJ-dependent mechanisms. Additionally, it has been shown that the re-expression of Cx43 in mammary carcinoma cell lines lacking endogenous Cx43 enabled the formation of heterotypic GJIC with microvascular endothelial cells and thus increased their diapedesis (177). Moreover, Cx43-GJICs between breast cancer cells and endothelial cells facilitate the metastatic homing of the tumor cells by increasing their arrest in the lung vasculature (178). Interestingly, the co-administration of avastatin (an anti-VEGF antibody used for anti-angiogenic therapy) and oleamide (a GJ chemical inhibitor) or even the administration of oleamide alone, decreases the heterotypic cell communications between MDA-MB-231 breast cancer cells with endothelial cells *in vitro*, increases their survival rate, and reduces pulmonary and hepatic metastatic foci in mice subdermally injected with MDA-MB-231 cells (179). However, the *in vivo* administration of oleamide alone does not inhibit metastasis to the lung in mice intravenously injected with MDA-MB-231 cells, suggesting that the inhibition of breast tumor-endothelial cell GJs has an anti-metastatic activity at the extravasation level (179).

Furthermore, it has been shown that primary and metastatic tumor cells can differentially modulate the expression of Cx proteins in endothelial cells. The heat-shock protein 27 released from cells derived from a primary colon tumor induces both the phosphorylation of Cx43 in endothelial cells and the formation of GJs among tumor and endothelial cells, promoting the transendothelial migration of malignant cells (180). In contrast, cells derived from a metastatic colon tumor from the same patient, induce the expression of Cx32-HCs in endothelial cells *via* CXCR2. The subsequent release of ATP through the Cx32-HCs by endothelial cells then modulates the crosstalk between endothelial and metastatic colon cancer cells, possibly favoring neo-angiogenesis in the metastatic foci (180). Similarly, glioblastom multiform (GBM) cells can also modulate endothelial cell function through heterotypic GJICs. The GJ-mediated transfer of the miRNA miR-5096 from GBM cells to endothelial cells promotes endothelial tubulogenesis by increasing the expression of Cx43 and the concomitant formation of heterotypic GJICs (181). Heterotypic GJs have also been described between leukemic and endothelial cells, allowing cancer cell migration and extravasation (182, 183). Finally, lung carcinoma and gastric cancer cells use heterotypic Cx43-GJICs with lymphatic endothelial cells and peritoneal mesothelial cells to support their migration through the lymphatic endothelium or the peritoneal mesothelium, respectively (184, 185). These evidences suggest that tumor cells engage GJICs with endothelial and epithelial cells to promote their migration, invasion and metastasis *via* blood vessels, lymphatic endothelium and peritoneal mesothelium. Taking into account the evidences described so far, we can speculate that the expression of Cxs by tumor cells growing in a vascularized microenvironment could be considered as a negative prognosis marker in cancer. However, as we previously discussed, GJICs among tumor cells and endothelial cells could also allow the cross-recognition and elimination of endothelial cells by tumor-Ag-specific CTLs (138). Therefore, we propose that Cx expression by tumor cells in a vascularized microenvironment could have a negative impact in patients with tumors poorly infiltrated by CTLs or a positive effect in those with tumors highly infiltrated by CTL; however, this hypothesis needs to be addressed.

Additionally, pro-tumoral GJICs among BMSCs and malignant cells have also been described. Reports from different groups have shown that GJICs between BMSCs and leukemic or breast cancer cells mediate the cell cycle quiescence of tumor cells. Leukemic cells coupled with BMSCs are arrested in G0, and these coupled leukemic cells are resistant to methotrexate-induced apoptosis, which can be prevented with treatments with the GJ inhibitor carbenoxolone (186). Similarly, Lim and collaborators reported that breast cancer cells acquired CXCL12-specific miRNAs from BMSCs *via* GJs, which is associated with the maintenance of cancer cell dormancy (187). These studies partly show how metastatic tumor cells could take advantage of GJ coupling with the bone marrow microenvironment for their survival.

The GJs among tumor cells and astrocytes are another example of pro-tumoral heterotypic GJICs occurring in cancer. Astrocytes are the most abundant glial cell population of the central nervous system (CNS), and they participate in the local innate immune response triggered by a variety of insults (188). Indeed, the majority of cancer cells that infiltrate the brain are eliminated by astrocytes (189). However, astrocytes can exert a beneficial effect on cancer cells through GJ-dependent manners (Figure 1, left panel). GJICs between GBM cells and astrocytes contribute somehow to the adhesion, migration and invasion of tumor cells to the brain parenchyma (190, 191). Recent evidence suggests that Cx43 expression in glioma cells and astrocytes influences tumor cell motility *in vivo* independently of its channel function (192). In a very elegant report by Chen and coworkers (110), it was demonstrated that protocadherin 7, which is expressed in human and mouse breast and lung cancer cells, promotes the assembly of Cx43-GJs between carcinoma cells and astrocytes. These Cx43-GJs allow metastatic cancer cells in the brain to transfer cGAMP to astrocytes, leading to the activation of the STING pathway and the subsequent production of IFN-α and TNF by the cGAMP-receiving astrocytes. These pro-inflammatory cytokines then cause paracrine activation of the STAT1 and NF-κB pathways in brain metastatic cells, supporting tumor growth and chemoresistance. Interestingly, *in vivo* inhibition of carcinoma-astrocyte GJICs, through the oral delivery of meclofenamate and tonabersat, blocks this paracrine loop, controlling metastatic outgrowth in the brain (110). Moreover, it has been shown that astrocytes can protect tumor cells from chemotherapy through additional GJ-mediated mechanisms. For example, reactive astrocytes reduce apoptosis in melanoma cells treated with different chemotherapeutic drugs by sequestering intracellular calcium *via* GJICs (104). In addition, astrocytes seem to upregulate the expression of various pro-survival genes in glioma cells trough a GJ-dependent manner, thus reducing the cytotoxic effects of various chemotherapeutic agents in tumor cells (193). Recently, it was shown that lung cancer cells acquire miRNAs from astrocytes in a GJ-dependent manner during *in vitro* cocultures (111). Several of the transferred miRNAs were implicated in cell survival pathways, and the enforced expression of these miRNAs increases the resistance of lung cancer cells to paclitaxel (111). Similarly, the GJ-mediated transfer of miR-5096 from glioma cells to astrocytes induces *in vitro* glioma cell invasion (194). Altogether, these data suggest that GJICs occurring between tumor cells and the main immune cells of the CNS, namely astrocytes, allow for the intercellular passage of signals that promote the colonization and survival of tumor cells in the brain.

These exciting and promising new evidences in our understanding of GJICs among tumor cells and their surrounding stromal cells, and particularly immune cells, generates the idea of potentiating the antitumor immune responses induced by current cancer immunotherapies *via* the modulation of GJIC. The implementation of this concept absolutely deserves further attention.

## Concluding Remarks

The essential role of GJICs among tumor cells and neighboring cells of the tumor microenvironment, including immune cells, on tumor cell fate and their relationship with cancer progression is only beginning to be understood. The current literature about cell intrinsic mechanisms of Cxs and homotypic GJICs among tumor cells, in general terms, would support a tumor suppressor role of Cxs in early stages of cancer progression, while in late-stage cancer and metastasis, Cxs could act as oncogenes, promoting the progression of cancer. It is worth noting that this general conclusion depends on the Cx isoforms expressed as well as on the tumor type or subtype. Additionally, the different heterotypic GJICs occurring in the tumor microenvironment should be included in this panorama. For example, while heterotypic GJICs among tumor cells and astrocytes seem to promote tumor progression, heterotypic GJICs between tumor cells and DCs or lymphocytes are involved in tumor cell elimination. Accordingly, re-assessing Cx expression along with a deep characterization of immune cell infiltration in human tumors may, in our opinion, definitively solve the complexity of the mixed findings related to Cxs as a useful diagnostic method (Table 1). Precision-medicine diagnostic tools, such as multilabel immunofluorescence on formalin-fixed paraffin-embedded sections (195) are suitable for evaluation of Cx expression and localization in different cells from the tumor microenvironment. Although much of the data discussed in this review come from *in vivo* studies, several of the most exciting findings remains to be validated on accurate and specific physiological models. Smart experimental designs using current murine models, such as conditional knockout of Cx43 in T cells (76) or in DCs (127), are required to fully elucidate the physiopathological implication of GJICs on tumor immunity. Additionally, major efforts must be made to determine which intercellular signals are involved in GJ-mediated tumor immunity. In our opinion, special focus should be put on the identification and characterization of immune modulatory miRNAs that can be transferred between tumor and immune cells, and therefore, affect tumor immune attack and/or tumor immune escape.

Nowadays, novel Cx and GJ-based therapeutic approaches have emerged, particularly based on small peptides that specifically block Cx-HCs or enhance GJ plaque formation. These therapeutic approaches have shown promising results in the context of cutaneous wound healing, tissue regeneration, narcolepsy, and reperfusion injury after acute myocardial infarction (196). Efforts to move these therapeutic approaches to cancer should be made in order to promote GJ-mediated antitumor immunity. It seems clear that targeting GJIC alone is not likely to be sufficient and combination with immunotherapy treatments will be necessary.

## Author Contributions

MG, MN, FH, FS-O, and AT contributed to the writing of the manuscript; AT contributed to the conception of figures and tables.

## Conflict of Interest Statement

The authors declare that the research was conducted in the absence of any commercial or financial relationships that could be construed as a potential conflict of interest.
